# Supplementary material for: Pyruvate Dehydrogenase Kinase 1 inhibition mediated oxidative phosphorylation enhancement in cartilage promotes osteoarthritis progression
Source: BMC Musculoskelet Disord. 2023 Jul 20;24:597. doi: 10.1186/s12891-023-06585-6 (PMC10357736; doi:10.1186/s12891-023-06585-6)
Supplement: Supplementary file 1 — Additional file 1. [file 12891_2023_6585_MOESM1_ESM.zip › MF.Gsea.1653627316242/GOMF_GTPASE_ACTIVATOR_ACTIVITY.html]

Details for gene set GOMF\_GTPASE\_ACTIVATOR\_ACTIVITY[GSEA]

|  || Dataset | 55457\_collapsed\_to\_symbols.cls.cls #OA\_versus\_normal.cls.cls #OA\_versus\_normal\_repos |
| Phenotype | cls.cls#OA\_versus\_normal\_repos |
| Upregulated in class | normal |
| GeneSet | GOMF\_GTPASE\_ACTIVATOR\_ACTIVITY |
| Enrichment Score (ES) | -0.29493704 |
| Normalized Enrichment Score (NES) | -1.4175674 |
| Nominal p-value | 0.008746356 |
| FDR q-value | 0.25680012 |
| FWER p-Value | 1.0 |
Table: GSEA Results Summary

  

Fig 1: Enrichment plot: GOMF\_GTPASE\_ACTIVATOR\_ACTIVITY      
 Profile of the Running ES Score & Positions of GeneSet Members on the Rank Ordered List

  

| SYMBOL | TITLE | RANK IN GENE LIST | RANK METRIC SCORE | RUNNING ES | CORE ENRICHMENT || 1 | ADGRB3 | adhesion G protein-coupled receptor B3 [Source:HGNC Symbol;Acc:HGNC:945] | 131 | 0.874 | 0.0044 | No |
| 2 | RALBP1 | ralA binding protein 1 [Source:HGNC Symbol;Acc:HGNC:9841] | 217 | 0.808 | 0.0113 | No |
| 3 | TBC1D4 | TBC1 domain family member 4 [Source:HGNC Symbol;Acc:HGNC:19165] | 320 | 0.737 | 0.0157 | No |
| 4 | NPRL2 | "NPR2 like, GATOR1 complex subunit [Source:HGNC Symbol;Acc:HGNC:24969]" | 429 | 0.695 | 0.0189 | No |
| 5 | RGS4 | regulator of G protein signaling 4 [Source:HGNC Symbol;Acc:HGNC:10000] | 449 | 0.683 | 0.0289 | No |
| 6 | DOCK2 | dedicator of cytokinesis 2 [Source:HGNC Symbol;Acc:HGNC:2988] | 520 | 0.652 | 0.0344 | No |
| 7 | TBC1D19 | TBC1 domain family member 19 [Source:HGNC Symbol;Acc:HGNC:25624] | 528 | 0.648 | 0.0447 | No |
| 8 | HTR2B | 5-hydroxytryptamine receptor 2B [Source:HGNC Symbol;Acc:HGNC:5294] | 562 | 0.636 | 0.0529 | No |
| 9 | RGS10 | regulator of G protein signaling 10 [Source:HGNC Symbol;Acc:HGNC:9992] | 635 | 0.610 | 0.0575 | No |
| 10 | SMAP1 | small ArfGAP 1 [Source:HGNC Symbol;Acc:HGNC:19651] | 1243 | 0.468 | 0.0176 | No |
| 11 | RGS20 | regulator of G protein signaling 20 [Source:HGNC Symbol;Acc:HGNC:14600] | 1345 | 0.451 | 0.0172 | No |
| 12 | ASAP3 | "ArfGAP with SH3 domain, ankyrin repeat and PH domain 3 [Source:HGNC Symbol;Acc:HGNC:14987]" | 1480 | 0.432 | 0.0139 | No |
| 13 | SIPA1 | signal-induced proliferation-associated 1 [Source:HGNC Symbol;Acc:HGNC:10885] | 1488 | 0.431 | 0.0206 | No |
| 14 | ADAP1 | ArfGAP with dual PH domains 1 [Source:HGNC Symbol;Acc:HGNC:16486] | 1706 | 0.398 | 0.0102 | No |
| 15 | TBC1D22A | TBC1 domain family member 22A [Source:HGNC Symbol;Acc:HGNC:1309] | 1718 | 0.395 | 0.0160 | No |
| 16 | ALDH1A1 | aldehyde dehydrogenase 1 family member A1 [Source:HGNC Symbol;Acc:HGNC:402] | 1737 | 0.393 | 0.0212 | No |
| 17 | RAB3GAP1 | RAB3 GTPase activating protein catalytic subunit 1 [Source:HGNC Symbol;Acc:HGNC:17063] | 1766 | 0.389 | 0.0256 | No |
| 18 | ARHGAP25 | Rho GTPase activating protein 25 [Source:HGNC Symbol;Acc:HGNC:28951] | 1900 | 0.373 | 0.0214 | No |
| 19 | TBC1D5 | TBC1 domain family member 5 [Source:HGNC Symbol;Acc:HGNC:19166] | 1956 | 0.367 | 0.0232 | No |
| 20 | USP6NL | USP6 N-terminal like [Source:HGNC Symbol;Acc:HGNC:16858] | 1999 | 0.361 | 0.0260 | No |
| 21 | ARHGAP24 | Rho GTPase activating protein 24 [Source:HGNC Symbol;Acc:HGNC:25361] | 2040 | 0.357 | 0.0289 | No |
| 22 | ARRB1 | arrestin beta 1 [Source:HGNC Symbol;Acc:HGNC:711] | 2116 | 0.348 | 0.0288 | No |
| 23 | RABGAP1L | RAB GTPase activating protein 1 like [Source:HGNC Symbol;Acc:HGNC:24663] | 2334 | 0.322 | 0.0172 | No |
| 24 | TBC1D2B | TBC1 domain family member 2B [Source:HGNC Symbol;Acc:HGNC:29183] | 2375 | 0.317 | 0.0193 | No |
| 25 | ARHGAP15 | Rho GTPase activating protein 15 [Source:HGNC Symbol;Acc:HGNC:21030] | 2420 | 0.312 | 0.0211 | No |
| 26 | RALGAPB | Ral GTPase activating protein non-catalytic subunit beta [Source:HGNC Symbol;Acc:HGNC:29221] | 2426 | 0.311 | 0.0260 | No |
| 27 | RASA1 | RAS p21 protein activator 1 [Source:HGNC Symbol;Acc:HGNC:9871] | 2506 | 0.302 | 0.0249 | No |
| 28 | TBC1D16 | TBC1 domain family member 16 [Source:HGNC Symbol;Acc:HGNC:28356] | 2536 | 0.299 | 0.0276 | No |
| 29 | ARFGAP2 | ADP ribosylation factor GTPase activating protein 2 [Source:HGNC Symbol;Acc:HGNC:13504] | 2548 | 0.297 | 0.0317 | No |
| 30 | NCKAP1L | NCK associated protein 1 like [Source:HGNC Symbol;Acc:HGNC:4862] | 2560 | 0.296 | 0.0359 | No |
| 31 | STARD8 | StAR related lipid transfer domain containing 8 [Source:HGNC Symbol;Acc:HGNC:19161] | 2785 | 0.271 | 0.0228 | No |
| 32 | GMIP | GEM interacting protein [Source:HGNC Symbol;Acc:HGNC:24852] | 2835 | 0.267 | 0.0234 | No |
| 33 | RGS7 | regulator of G protein signaling 7 [Source:HGNC Symbol;Acc:HGNC:10003] | 2842 | 0.266 | 0.0274 | No |
| 34 | FAM13A | family with sequence similarity 13 member A [Source:HGNC Symbol;Acc:HGNC:19367] | 3048 | 0.245 | 0.0154 | No |
| 35 | RANBP1 | RAN binding protein 1 [Source:HGNC Symbol;Acc:HGNC:9847] | 3214 | 0.229 | 0.0063 | No |
| 36 | RGS3 | regulator of G protein signaling 3 [Source:HGNC Symbol;Acc:HGNC:9999] | 3247 | 0.226 | 0.0076 | No |
| 37 | TBC1D9 | TBC1 domain family member 9 [Source:HGNC Symbol;Acc:HGNC:21710] | 3287 | 0.222 | 0.0082 | No |
| 38 | PLEKHG6 | pleckstrin homology and RhoGEF domain containing G6 [Source:HGNC Symbol;Acc:HGNC:25562] | 3662 | 0.189 | -0.0180 | No |
| 39 | NRP1 | neuropilin 1 [Source:HGNC Symbol;Acc:HGNC:8004] | 3704 | 0.185 | -0.0181 | No |
| 40 | PLCB1 | phospholipase C beta 1 [Source:HGNC Symbol;Acc:HGNC:15917] | 3724 | 0.183 | -0.0165 | No |
| 41 | SEC23B | "SEC23 homolog B, COPII coat complex component [Source:HGNC Symbol;Acc:HGNC:10702]" | 3838 | 0.172 | -0.0225 | No |
| 42 | SEC23A | "SEC23 homolog A, COPII coat complex component [Source:HGNC Symbol;Acc:HGNC:10701]" | 4017 | 0.154 | -0.0340 | No |
| 43 | TBC1D8B | TBC1 domain family member 8B [Source:HGNC Symbol;Acc:HGNC:24715] | 4040 | 0.152 | -0.0331 | No |
| 44 | ARHGDIG | Rho GDP dissociation inhibitor gamma [Source:HGNC Symbol;Acc:HGNC:680] | 4063 | 0.150 | -0.0323 | No |
| 45 | VAV3 | vav guanine nucleotide exchange factor 3 [Source:HGNC Symbol;Acc:HGNC:12659] | 4082 | 0.148 | -0.0313 | No |
| 46 | ARHGDIB | Rho GDP dissociation inhibitor beta [Source:HGNC Symbol;Acc:HGNC:679] | 4085 | 0.147 | -0.0289 | No |
| 47 | TBC1D1 | TBC1 domain family member 1 [Source:HGNC Symbol;Acc:HGNC:11578] | 4215 | 0.136 | -0.0368 | No |
| 48 | RAP1GDS1 | Rap1 GTPase-GDP dissociation stimulator 1 [Source:HGNC Symbol;Acc:HGNC:9859] | 4234 | 0.134 | -0.0360 | No |
| 49 | RGS11 | regulator of G protein signaling 11 [Source:HGNC Symbol;Acc:HGNC:9993] | 4304 | 0.128 | -0.0392 | No |
| 50 | ARHGAP11A | Rho GTPase activating protein 11A [Source:HGNC Symbol;Acc:HGNC:15783] | 4326 | 0.126 | -0.0388 | No |
| 51 | ARHGAP45 | Rho GTPase activating protein 45 [Source:HGNC Symbol;Acc:HGNC:17102] | 4402 | 0.121 | -0.0426 | No |
| 52 | DEPDC1 | DEP domain containing 1 [Source:HGNC Symbol;Acc:HGNC:22949] | 4482 | 0.114 | -0.0469 | No |
| 53 | LLGL2 | LLGL scribble cell polarity complex component 2 [Source:HGNC Symbol;Acc:HGNC:6629] | 4604 | 0.104 | -0.0547 | No |
| 54 | DOCK4 | dedicator of cytokinesis 4 [Source:HGNC Symbol;Acc:HGNC:19192] | 4607 | 0.104 | -0.0531 | No |
| 55 | ALS2CL | ALS2 C-terminal like [Source:HGNC Symbol;Acc:HGNC:20605] | 4668 | 0.099 | -0.0562 | No |
| 56 | WNT11 | Wnt family member 11 [Source:HGNC Symbol;Acc:HGNC:12776] | 4688 | 0.097 | -0.0560 | No |
| 57 | ARAP1 | "ArfGAP with RhoGAP domain, ankyrin repeat and PH domain 1 [Source:HGNC Symbol;Acc:HGNC:16925]" | 4730 | 0.093 | -0.0577 | No |
| 58 | TBC1D12 | TBC1 domain family member 12 [Source:HGNC Symbol;Acc:HGNC:29082] | 4931 | 0.078 | -0.0721 | No |
| 59 | THY1 | Thy-1 cell surface antigen [Source:HGNC Symbol;Acc:HGNC:11801] | 4985 | 0.074 | -0.0751 | No |
| 60 | DOCK1 | dedicator of cytokinesis 1 [Source:HGNC Symbol;Acc:HGNC:2987] | 4998 | 0.073 | -0.0748 | No |
| 61 | ACAP1 | "ArfGAP with coiled-coil, ankyrin repeat and PH domains 1 [Source:HGNC Symbol;Acc:HGNC:16467]" | 5210 | 0.056 | -0.0904 | No |
| 62 | ADAP2 | ArfGAP with dual PH domains 2 [Source:HGNC Symbol;Acc:HGNC:16487] | 5535 | 0.030 | -0.1154 | No |
| 63 | FAM13B | family with sequence similarity 13 member B [Source:HGNC Symbol;Acc:HGNC:1335] | 5589 | 0.025 | -0.1192 | No |
| 64 | ASAP2 | "ArfGAP with SH3 domain, ankyrin repeat and PH domain 2 [Source:HGNC Symbol;Acc:HGNC:2721]" | 5742 | 0.012 | -0.1310 | No |
| 65 | HACD3 | 3-hydroxyacyl-CoA dehydratase 3 [Source:HGNC Symbol;Acc:HGNC:24175] | 5755 | 0.011 | -0.1317 | No |
| 66 | ARHGAP4 | Rho GTPase activating protein 4 [Source:HGNC Symbol;Acc:HGNC:674] | 6002 | -0.004 | -0.1510 | No |
| 67 | PREB | prolactin regulatory element binding [Source:HGNC Symbol;Acc:HGNC:9356] | 6044 | -0.008 | -0.1541 | No |
| 68 | BNIP2 | BCL2 interacting protein 2 [Source:HGNC Symbol;Acc:HGNC:1083] | 6045 | -0.008 | -0.1540 | No |
| 69 | SYDE1 | synapse defective Rho GTPase homolog 1 [Source:HGNC Symbol;Acc:HGNC:25824] | 6066 | -0.009 | -0.1554 | No |
| 70 | ARHGAP12 | Rho GTPase activating protein 12 [Source:HGNC Symbol;Acc:HGNC:16348] | 6099 | -0.013 | -0.1577 | No |
| 71 | SIPA1L1 | signal induced proliferation associated 1 like 1 [Source:HGNC Symbol;Acc:HGNC:20284] | 6151 | -0.016 | -0.1614 | No |
| 72 | TBC1D30 | TBC1 domain family member 30 [Source:HGNC Symbol;Acc:HGNC:29164] | 6231 | -0.023 | -0.1673 | No |
| 73 | RASAL1 | RAS protein activator like 1 [Source:HGNC Symbol;Acc:HGNC:9873] | 6337 | -0.031 | -0.1750 | No |
| 74 | GDI2 | GDP dissociation inhibitor 2 [Source:HGNC Symbol;Acc:HGNC:4227] | 6525 | -0.044 | -0.1890 | No |
| 75 | LLGL1 | LLGL scribble cell polarity complex component 1 [Source:HGNC Symbol;Acc:HGNC:6628] | 6528 | -0.044 | -0.1884 | No |
| 76 | RIN2 | Ras and Rab interactor 2 [Source:HGNC Symbol;Acc:HGNC:18750] | 6538 | -0.045 | -0.1884 | No |
| 77 | CHN1 | chimerin 1 [Source:HGNC Symbol;Acc:HGNC:1943] | 6563 | -0.047 | -0.1895 | No |
| 78 | ARHGEF12 | Rho guanine nucleotide exchange factor 12 [Source:HGNC Symbol;Acc:HGNC:14193] | 6587 | -0.049 | -0.1905 | No |
| 79 | PREX2 | "phosphatidylinositol-3,4,5-trisphosphate dependent Rac exchange factor 2 [Source:HGNC Symbol;Acc:HGNC:22950]" | 6632 | -0.052 | -0.1930 | No |
| 80 | ARHGAP22 | Rho GTPase activating protein 22 [Source:HGNC Symbol;Acc:HGNC:30320] | 6657 | -0.053 | -0.1940 | No |
| 81 | ARHGDIA | Rho GDP dissociation inhibitor alpha [Source:HGNC Symbol;Acc:HGNC:678] | 6664 | -0.053 | -0.1936 | No |
| 82 | RGS6 | regulator of G protein signaling 6 [Source:HGNC Symbol;Acc:HGNC:10002] | 6706 | -0.056 | -0.1959 | No |
| 83 | RIN1 | Ras and Rab interactor 1 [Source:HGNC Symbol;Acc:HGNC:18749] | 6730 | -0.058 | -0.1967 | No |
| 84 | ANKRD27 | ankyrin repeat domain 27 [Source:HGNC Symbol;Acc:HGNC:25310] | 6770 | -0.061 | -0.1988 | No |
| 85 | TSC2 | TSC complex subunit 2 [Source:HGNC Symbol;Acc:HGNC:12363] | 6946 | -0.075 | -0.2113 | No |
| 86 | ARAP2 | "ArfGAP with RhoGAP domain, ankyrin repeat and PH domain 2 [Source:HGNC Symbol;Acc:HGNC:16924]" | 6983 | -0.079 | -0.2128 | No |
| 87 | DLC1 | DLC1 Rho GTPase activating protein [Source:HGNC Symbol;Acc:HGNC:2897] | 6984 | -0.079 | -0.2115 | No |
| 88 | ARHGAP28 | Rho GTPase activating protein 28 [Source:HGNC Symbol;Acc:HGNC:25509] | 7005 | -0.080 | -0.2117 | No |
| 89 | RANBP3 | RAN binding protein 3 [Source:HGNC Symbol;Acc:HGNC:9850] | 7043 | -0.083 | -0.2132 | No |
| 90 | AGAP1 | "ArfGAP with GTPase domain, ankyrin repeat and PH domain 1 [Source:HGNC Symbol;Acc:HGNC:16922]" | 7081 | -0.086 | -0.2147 | No |
| 91 | SRGAP3 | SLIT-ROBO Rho GTPase activating protein 3 [Source:HGNC Symbol;Acc:HGNC:19744] | 7091 | -0.087 | -0.2139 | No |
| 92 | RIN3 | Ras and Rab interactor 3 [Source:HGNC Symbol;Acc:HGNC:18751] | 7110 | -0.089 | -0.2138 | No |
| 93 | GNB5 | G protein subunit beta 5 [Source:HGNC Symbol;Acc:HGNC:4401] | 7223 | -0.097 | -0.2210 | No |
| 94 | MTSS2 | MTSS I-BAR domain containing 2 [Source:HGNC Symbol;Acc:HGNC:25094] | 7240 | -0.098 | -0.2206 | No |
| 95 | TBC1D13 | TBC1 domain family member 13 [Source:HGNC Symbol;Acc:HGNC:25571] | 7261 | -0.100 | -0.2205 | No |
| 96 | TBC1D9B | TBC1 domain family member 9B [Source:HGNC Symbol;Acc:HGNC:29097] | 7263 | -0.100 | -0.2189 | No |
| 97 | RACGAP1 | Rac GTPase activating protein 1 [Source:HGNC Symbol;Acc:HGNC:9804] | 7269 | -0.100 | -0.2176 | No |
| 98 | OCRL | OCRL inositol polyphosphate-5-phosphatase [Source:HGNC Symbol;Acc:HGNC:8108] | 7319 | -0.103 | -0.2197 | No |
| 99 | ARHGAP5 | Rho GTPase activating protein 5 [Source:HGNC Symbol;Acc:HGNC:675] | 7357 | -0.106 | -0.2208 | No |
| 100 | TBC1D2 | TBC1 domain family member 2 [Source:HGNC Symbol;Acc:HGNC:18026] | 7399 | -0.111 | -0.2222 | No |
| 101 | GNAQ | G protein subunit alpha q [Source:HGNC Symbol;Acc:HGNC:4390] | 7518 | -0.120 | -0.2295 | No |
| 102 | EVI5 | ecotropic viral integration site 5 [Source:HGNC Symbol;Acc:HGNC:3501] | 7616 | -0.128 | -0.2350 | No |
| 103 | RGS14 | regulator of G protein signaling 14 [Source:HGNC Symbol;Acc:HGNC:9996] | 7719 | -0.135 | -0.2407 | No |
| 104 | AGAP2 | "ArfGAP with GTPase domain, ankyrin repeat and PH domain 2 [Source:HGNC Symbol;Acc:HGNC:16921]" | 7732 | -0.136 | -0.2394 | No |
| 105 | AGFG2 | ArfGAP with FG repeats 2 [Source:HGNC Symbol;Acc:HGNC:5177] | 7796 | -0.141 | -0.2419 | No |
| 106 | CHN2 | chimerin 2 [Source:HGNC Symbol;Acc:HGNC:1944] | 7847 | -0.144 | -0.2435 | No |
| 107 | ARHGEF6 | Rac/Cdc42 guanine nucleotide exchange factor 6 [Source:HGNC Symbol;Acc:HGNC:685] | 7900 | -0.147 | -0.2451 | No |
| 108 | RAP1GAP | RAP1 GTPase activating protein [Source:HGNC Symbol;Acc:HGNC:9858] | 7910 | -0.148 | -0.2433 | No |
| 109 | RAP1GAP2 | RAP1 GTPase activating protein 2 [Source:HGNC Symbol;Acc:HGNC:29176] | 7955 | -0.152 | -0.2442 | No |
| 110 | RAB3GAP2 | RAB3 GTPase activating non-catalytic protein subunit 2 [Source:HGNC Symbol;Acc:HGNC:17168] | 7957 | -0.152 | -0.2417 | No |
| 111 | SRGAP2 | SLIT-ROBO Rho GTPase activating protein 2 [Source:HGNC Symbol;Acc:HGNC:19751] | 8116 | -0.164 | -0.2514 | No |
| 112 | RABEP1 | "rabaptin, RAB GTPase binding effector protein 1 [Source:HGNC Symbol;Acc:HGNC:17677]" | 8165 | -0.168 | -0.2523 | No |
| 113 | ABR | ABR activator of RhoGEF and GTPase [Source:HGNC Symbol;Acc:HGNC:81] | 8184 | -0.170 | -0.2509 | No |
| 114 | TBC1D15 | TBC1 domain family member 15 [Source:HGNC Symbol;Acc:HGNC:25694] | 8197 | -0.171 | -0.2490 | No |
| 115 | TBC1D17 | TBC1 domain family member 17 [Source:HGNC Symbol;Acc:HGNC:25699] | 8230 | -0.173 | -0.2486 | No |
| 116 | STXBP5L | syntaxin binding protein 5L [Source:HGNC Symbol;Acc:HGNC:30757] | 8240 | -0.174 | -0.2463 | No |
| 117 | IQGAP1 | IQ motif containing GTPase activating protein 1 [Source:HGNC Symbol;Acc:HGNC:6110] | 8276 | -0.177 | -0.2461 | No |
| 118 | ARHGAP17 | Rho GTPase activating protein 17 [Source:HGNC Symbol;Acc:HGNC:18239] | 8325 | -0.180 | -0.2469 | No |
| 119 | DEPDC5 | "DEP domain containing 5, GATOR1 subcomplex subunit [Source:HGNC Symbol;Acc:HGNC:18423]" | 8331 | -0.180 | -0.2442 | No |
| 120 | ARHGAP32 | Rho GTPase activating protein 32 [Source:HGNC Symbol;Acc:HGNC:17399] | 8370 | -0.183 | -0.2441 | No |
| 121 | DOCK5 | dedicator of cytokinesis 5 [Source:HGNC Symbol;Acc:HGNC:23476] | 8380 | -0.184 | -0.2417 | No |
| 122 | DNM1L | dynamin 1 like [Source:HGNC Symbol;Acc:HGNC:2973] | 8499 | -0.194 | -0.2478 | No |
| 123 | TBC1D22B | TBC1 domain family member 22B [Source:HGNC Symbol;Acc:HGNC:21602] | 8571 | -0.199 | -0.2500 | No |
| 124 | IQGAP2 | IQ motif containing GTPase activating protein 2 [Source:HGNC Symbol;Acc:HGNC:6111] | 8598 | -0.201 | -0.2487 | No |
| 125 | ECT2 | epithelial cell transforming 2 [Source:HGNC Symbol;Acc:HGNC:3155] | 8647 | -0.204 | -0.2490 | No |
| 126 | ARHGAP35 | Rho GTPase activating protein 35 [Source:HGNC Symbol;Acc:HGNC:4591] | 8835 | -0.220 | -0.2600 | No |
| 127 | FLCN | folliculin [Source:HGNC Symbol;Acc:HGNC:27310] | 8848 | -0.221 | -0.2572 | No |
| 128 | ARHGEF10L | Rho guanine nucleotide exchange factor 10 like [Source:HGNC Symbol;Acc:HGNC:25540] | 9020 | -0.233 | -0.2668 | No |
| 129 | RASA2 | RAS p21 protein activator 2 [Source:HGNC Symbol;Acc:HGNC:9872] | 9153 | -0.244 | -0.2731 | No |
| 130 | RASAL2 | RAS protein activator like 2 [Source:HGNC Symbol;Acc:HGNC:9874] | 9161 | -0.245 | -0.2695 | No |
| 131 | NF1 | neurofibromin 1 [Source:HGNC Symbol;Acc:HGNC:7765] | 9194 | -0.247 | -0.2678 | No |
| 132 | GIT1 | GIT ArfGAP 1 [Source:HGNC Symbol;Acc:HGNC:4272] | 9236 | -0.251 | -0.2668 | No |
| 133 | ARHGAP1 | Rho GTPase activating protein 1 [Source:HGNC Symbol;Acc:HGNC:673] | 9307 | -0.256 | -0.2680 | No |
| 134 | RGS5 | regulator of G protein signaling 5 [Source:HGNC Symbol;Acc:HGNC:10001] | 9432 | -0.267 | -0.2733 | No |
| 135 | GIT2 | GIT ArfGAP 2 [Source:HGNC Symbol;Acc:HGNC:4273] | 9552 | -0.278 | -0.2780 | No |
| 136 | RASGRP3 | RAS guanyl releasing protein 3 [Source:HGNC Symbol;Acc:HGNC:14545] | 9603 | -0.284 | -0.2771 | No |
| 137 | BCR | BCR activator of RhoGEF and GTPase [Source:HGNC Symbol;Acc:HGNC:1014] | 9830 | -0.304 | -0.2898 | Yes |
| 138 | TBC1D10B | TBC1 domain family member 10B [Source:HGNC Symbol;Acc:HGNC:24510] | 9880 | -0.310 | -0.2885 | Yes |
| 139 | ARHGAP29 | Rho GTPase activating protein 29 [Source:HGNC Symbol;Acc:HGNC:30207] | 9916 | -0.313 | -0.2859 | Yes |
| 140 | RABGAP1 | RAB GTPase activating protein 1 [Source:HGNC Symbol;Acc:HGNC:17155] | 9928 | -0.315 | -0.2815 | Yes |
| 141 | GRTP1 | growth hormone regulated TBC protein 1 [Source:HGNC Symbol;Acc:HGNC:20310] | 9930 | -0.315 | -0.2763 | Yes |
| 142 | SH3BP1 | SH3 domain binding protein 1 [Source:HGNC Symbol;Acc:HGNC:10824] | 10016 | -0.323 | -0.2776 | Yes |
| 143 | ARHGEF11 | Rho guanine nucleotide exchange factor 11 [Source:HGNC Symbol;Acc:HGNC:14580] | 10056 | -0.326 | -0.2751 | Yes |
| 144 | RGS12 | regulator of G protein signaling 12 [Source:HGNC Symbol;Acc:HGNC:9994] | 10163 | -0.337 | -0.2778 | Yes |
| 145 | STARD13 | StAR related lipid transfer domain containing 13 [Source:HGNC Symbol;Acc:HGNC:19164] | 10183 | -0.339 | -0.2736 | Yes |
| 146 | TBCD | tubulin folding cofactor D [Source:HGNC Symbol;Acc:HGNC:11581] | 10252 | -0.346 | -0.2731 | Yes |
| 147 | ASAP1 | "ArfGAP with SH3 domain, ankyrin repeat and PH domain 1 [Source:HGNC Symbol;Acc:HGNC:2720]" | 10276 | -0.349 | -0.2690 | Yes |
| 148 | RANGAP1 | Ran GTPase activating protein 1 [Source:HGNC Symbol;Acc:HGNC:9854] | 10288 | -0.349 | -0.2640 | Yes |
| 149 | RP2 | RP2 activator of ARL3 GTPase [Source:HGNC Symbol;Acc:HGNC:10274] | 10354 | -0.356 | -0.2631 | Yes |
| 150 | ARFGAP3 | ADP ribosylation factor GTPase activating protein 3 [Source:HGNC Symbol;Acc:HGNC:661] | 10382 | -0.359 | -0.2592 | Yes |
| 151 | SGSM2 | small G protein signaling modulator 2 [Source:HGNC Symbol;Acc:HGNC:29026] | 10403 | -0.361 | -0.2547 | Yes |
| 152 | GAPVD1 | GTPase activating protein and VPS9 domains 1 [Source:HGNC Symbol;Acc:HGNC:23375] | 10410 | -0.362 | -0.2491 | Yes |
| 153 | RGS1 | regulator of G protein signaling 1 [Source:HGNC Symbol;Acc:HGNC:9991] | 10422 | -0.363 | -0.2439 | Yes |
| 154 | ARFGEF1 | ADP ribosylation factor guanine nucleotide exchange factor 1 [Source:HGNC Symbol;Acc:HGNC:15772] | 10477 | -0.371 | -0.2419 | Yes |
| 155 | ARHGAP8 | Rho GTPase activating protein 8 [Source:HGNC Symbol;Acc:HGNC:677] | 10551 | -0.378 | -0.2412 | Yes |
| 156 | RABEP2 | "rabaptin, RAB GTPase binding effector protein 2 [Source:HGNC Symbol;Acc:HGNC:24817]" | 10568 | -0.380 | -0.2361 | Yes |
| 157 | RASA3 | RAS p21 protein activator 3 [Source:HGNC Symbol;Acc:HGNC:20331] | 10574 | -0.381 | -0.2301 | Yes |
| 158 | RGPD5 | RANBP2 like and GRIP domain containing 5 [Source:HGNC Symbol;Acc:HGNC:32418] | 10604 | -0.384 | -0.2259 | Yes |
| 159 | PLXNB1 | plexin B1 [Source:HGNC Symbol;Acc:HGNC:9103] | 10682 | -0.391 | -0.2254 | Yes |
| 160 | ARHGAP44 | Rho GTPase activating protein 44 [Source:HGNC Symbol;Acc:HGNC:29096] | 10733 | -0.397 | -0.2226 | Yes |
| 161 | ARFGAP1 | ADP ribosylation factor GTPase activating protein 1 [Source:HGNC Symbol;Acc:HGNC:15852] | 10827 | -0.408 | -0.2231 | Yes |
| 162 | MYO9B | myosin IXB [Source:HGNC Symbol;Acc:HGNC:7609] | 10864 | -0.412 | -0.2190 | Yes |
| 163 | SIPA1L3 | signal induced proliferation associated 1 like 3 [Source:HGNC Symbol;Acc:HGNC:23801] | 11036 | -0.435 | -0.2251 | Yes |
| 164 | RAPGEF2 | Rap guanine nucleotide exchange factor 2 [Source:HGNC Symbol;Acc:HGNC:16854] | 11041 | -0.435 | -0.2181 | Yes |
| 165 | RGS9 | regulator of G protein signaling 9 [Source:HGNC Symbol;Acc:HGNC:10004] | 11044 | -0.436 | -0.2109 | Yes |
| 166 | ARHGAP33 | Rho GTPase activating protein 33 [Source:HGNC Symbol;Acc:HGNC:23085] | 11130 | -0.447 | -0.2101 | Yes |
| 167 | ARHGAP6 | Rho GTPase activating protein 6 [Source:HGNC Symbol;Acc:HGNC:676] | 11187 | -0.456 | -0.2068 | Yes |
| 168 | ACAP2 | "ArfGAP with coiled-coil, ankyrin repeat and PH domains 2 [Source:HGNC Symbol;Acc:HGNC:16469]" | 11229 | -0.461 | -0.2023 | Yes |
| 169 | RGS17 | regulator of G protein signaling 17 [Source:HGNC Symbol;Acc:HGNC:14088] | 11249 | -0.463 | -0.1960 | Yes |
| 170 | ARHGEF15 | Rho guanine nucleotide exchange factor 15 [Source:HGNC Symbol;Acc:HGNC:15590] | 11489 | -0.500 | -0.2064 | Yes |
| 171 | TBC1D8 | TBC1 domain family member 8 [Source:HGNC Symbol;Acc:HGNC:17791] | 11490 | -0.500 | -0.1979 | Yes |
| 172 | VPS9D1 | VPS9 domain containing 1 [Source:HGNC Symbol;Acc:HGNC:13526] | 11495 | -0.501 | -0.1898 | Yes |
| 173 | USP6 | ubiquitin specific peptidase 6 [Source:HGNC Symbol;Acc:HGNC:12629] | 11570 | -0.511 | -0.1870 | Yes |
| 174 | RALGAPA1 | Ral GTPase activating protein catalytic subunit alpha 1 [Source:HGNC Symbol;Acc:HGNC:17770] | 11779 | -0.546 | -0.1942 | Yes |
| 175 | CHM | CHM Rab escort protein [Source:HGNC Symbol;Acc:HGNC:1940] | 11798 | -0.549 | -0.1864 | Yes |
| 176 | GDI1 | GDP dissociation inhibitor 1 [Source:HGNC Symbol;Acc:HGNC:4226] | 11803 | -0.550 | -0.1775 | Yes |
| 177 | ARHGAP19 | Rho GTPase activating protein 19 [Source:HGNC Symbol;Acc:HGNC:23724] | 11822 | -0.553 | -0.1696 | Yes |
| 178 | ARHGAP10 | Rho GTPase activating protein 10 [Source:HGNC Symbol;Acc:HGNC:26099] | 11998 | -0.592 | -0.1734 | Yes |
| 179 | TBC1D3F | TBC1 domain family member 3F [Source:HGNC Symbol;Acc:HGNC:18257] | 12019 | -0.597 | -0.1649 | Yes |
| 180 | AGFG1 | ArfGAP with FG repeats 1 [Source:HGNC Symbol;Acc:HGNC:5175] | 12035 | -0.602 | -0.1559 | Yes |
| 181 | RGS2 | regulator of G protein signaling 2 [Source:HGNC Symbol;Acc:HGNC:9998] | 12039 | -0.603 | -0.1460 | Yes |
| 182 | SOS1 | SOS Ras/Rac guanine nucleotide exchange factor 1 [Source:HGNC Symbol;Acc:HGNC:11187] | 12041 | -0.603 | -0.1359 | Yes |
| 183 | RASA4 | RAS p21 protein activator 4 [Source:HGNC Symbol;Acc:HGNC:23181] | 12099 | -0.619 | -0.1300 | Yes |
| 184 | CHML | CHM like Rab escort protein [Source:HGNC Symbol;Acc:HGNC:1941] | 12132 | -0.626 | -0.1220 | Yes |
| 185 | MYO9A | myosin IXA [Source:HGNC Symbol;Acc:HGNC:7608] | 12166 | -0.635 | -0.1139 | Yes |
| 186 | RASA4B | RAS p21 protein activator 4B [Source:HGNC Symbol;Acc:HGNC:35202] | 12265 | -0.667 | -0.1103 | Yes |
| 187 | ADPRH | ADP-ribosylarginine hydrolase [Source:HGNC Symbol;Acc:HGNC:269] | 12277 | -0.670 | -0.0999 | Yes |
| 188 | ARAP3 | "ArfGAP with RhoGAP domain, ankyrin repeat and PH domain 3 [Source:HGNC Symbol;Acc:HGNC:24097]" | 12386 | -0.704 | -0.0966 | Yes |
| 189 | LARS1 | leucyl-tRNA synthetase 1 [Source:HGNC Symbol;Acc:HGNC:6512] | 12415 | -0.712 | -0.0868 | Yes |
| 190 | ARHGAP26 | Rho GTPase activating protein 26 [Source:HGNC Symbol;Acc:HGNC:17073] | 12438 | -0.720 | -0.0764 | Yes |
| 191 | SGSM3 | small G protein signaling modulator 3 [Source:HGNC Symbol;Acc:HGNC:25228] | 12515 | -0.752 | -0.0697 | Yes |
| 192 | RGS16 | regulator of G protein signaling 16 [Source:HGNC Symbol;Acc:HGNC:9997] | 12523 | -0.759 | -0.0575 | Yes |
| 193 | NPRL3 | "NPR3 like, GATOR1 complex subunit [Source:HGNC Symbol;Acc:HGNC:14124]" | 12570 | -0.781 | -0.0479 | Yes |
| 194 | AGAP4 | "ArfGAP with GTPase domain, ankyrin repeat and PH domain 4 [Source:HGNC Symbol;Acc:HGNC:23459]" | 12608 | -0.803 | -0.0373 | Yes |
| 195 | RANBP2 | RAN binding protein 2 [Source:HGNC Symbol;Acc:HGNC:9848] | 12609 | -0.804 | -0.0238 | Yes |
| 196 | ARHGEF1 | Rho guanine nucleotide exchange factor 1 [Source:HGNC Symbol;Acc:HGNC:681] | 12673 | -0.846 | -0.0145 | Yes |
| 197 | CDC42EP2 | CDC42 effector protein 2 [Source:HGNC Symbol;Acc:HGNC:16263] | 12756 | -0.920 | -0.0055 | Yes |
| 198 | JUN | "Jun proto-oncogene, AP-1 transcription factor subunit [Source:HGNC Symbol;Acc:HGNC:6204]" | 12813 | -0.994 | 0.0068 | Yes |
Table: GSEA details [plain text format]

  

Fig 2: GOMF\_GTPASE\_ACTIVATOR\_ACTIVITY      
 Blue-Pink O' Gram in the Space of the Analyzed GeneSet

  

Fig 3: GOMF\_GTPASE\_ACTIVATOR\_ACTIVITY: Random ES distribution      
 Gene set null distribution of ES for **GOMF\_GTPASE\_ACTIVATOR\_ACTIVITY**
